# Amidoxime Modified UiO-66@PIM-1 Mixed-Matrix Membranes to Enhance CO_2_ Separation and Anti-Aging Performance

**DOI:** 10.3390/membranes13090781

**Published:** 2023-09-06

**Authors:** Jiaming Gao, Yongchao Sun, Feifei Kang, Fei Guo, Gaohong He, Hanli Wang, Zhendong Yang, Canghai Ma, Xiaobin Jiang, Wu Xiao

**Affiliations:** 1 State Key Laboratory of Fine Chemicals, Frontier Science Center for Smart Materials, Dalian University of Technology, Dalian 116024, China; jiaminggao97@outlook.com (J.G.); yongchao_sun@mail.dlut.edu.cn (Y.S.); kangfeifei1998@outlook.com (F.K.); guofei@mail.dlut.edu.cn (F.G.); cma@dlut.edu.cn (C.M.); xbjiang@dlut.edu.cn (X.J.); 2 Shandong Huaxia Shenzhou New Material Co., Ltd., Zibo 256401, China; whl89333@huaxiashenzhou.com (H.W.); yangzhendong@huaxiashenzhou.com (Z.Y.)

**Keywords:** amidoxime, UiO-66-AO, mixed matrix membrane, PIM-1, CO_2_/N_2_ separation

## Abstract

Mixed matrix membranes (MMMs) generally have some fatal defects, such as poor compatibility between the two phases leading to non-selective pores. In this work, PIM-1 was chosen as the polymer matrix, and UiO-66 modified with amidoxime (UiO-66-AO) was used as the filler to prepare the MMMs. In the MMMs, the amino and hydroxyl groups on UO-66-AO form a rich hydrogen bond network with the N and O atoms in the polymer PIM-1 chain to improve the compatibility between the polymer matrix and the filler. In addition, the selective adsorption of CO_2_ by the amidoxime group can promote the transport of CO_2_ in the membrane, which enhances the gas selectivity. The CO_2_ permeability and CO_2_/N_2_ selectivity of UiO-66-AO@PIM-1 MMMs are increased by 35.2% and 45.2% compared to pure PIM-1 membranes, reaching 7535.5 Barrer and 26.9, surpassing the Robeson Upper Bound (2008) and close to the 2019 Upper Bound. After 38 days of the aging experiment, the CO_2_ permeability is approximately 74% of the original. The results show that the addition of UiO-66-AO has an obvious effect on improving the aging properties of the membrane. The UiO-66-AO@PIM-1 MMMs have a bright prospect for CO_2_ separation in the future.

## 1. Introduction

The Intergovernmental Panel on Climate Change (IPCC) reported that greenhouse gases caused a global surface temperature increase of 0.4–1.3 °C from 1951–2010 [1]. It is predicted that by 2400, the increase in global greenhouse emissions will lead to a global average temperature increment of 2–6 °C. CO_2_ is responsible for about 26 percent of the total greenhouse effect. In this case, efficient carbon capture technologies are required to achieve carbon neutrality [2]. Currently, CO_2_ separation methods mainly focus on cryogenic separation [3], physical and chemical adsorption [4], absorption [5], and membrane separation technology [6]. Compared with traditional separation technologies, membrane separation has many advantages, such as low energy costs and being environmentally friendly during the process [7]. The small size of the membrane separation device saves more space for other devices, and its simple operation method makes it easier to operate. Consequently, membrane separation technology has gained much attention in CO_2_ separation. According to the membrane materials, CO_2_ separation membranes can be classified into polymer membranes [8], inorganic membranes [9], and mixed matrix membranes [10]. In addition to the trade-off effect between permeability and selectivity, plasticization and physical aging problems are unavoidable for polymer membranes [11,12]. Inorganic membranes are difficult to prepare, and the high cost of raw materials is a stumbling block in their industrialization [13]. Mixed matrix membranes are made of inorganic filler and polymer matrix, combining the advantages of each to achieve high gas separation performance as well as stability and economy [14].

Budd et al. [15] synthesized rigid, chain-like self-microporous polymers with twisted structures, PIM-1~PIM-6. Since the main chain of PIM-1 is not free to rotate, it can well hinder the limited stacking between molecular chains, resulting in the formation of continuous microporosity inside the PIM-1 membrane, which exhibits good CO_2_ permeability and moderate selectivity [16]. In addition, like other glassy polymers, the physical aging problems caused by intermolecular relaxation in the chain segments of PIM-1 have seriously hindered their commercial application. The blending of fillers into polymeric PIM-1 to prepare MMMs is a common means to break the Robeson upper bound. Meanwhile, the fillers can limit the free movement of polymer chains and significantly attenuate the physical aging problem of PIM membranes. The typical fillers in the MMM include metal-organic frameworks (MOFs) [17], zeolite [18], carbon molecular sieve [19], covalent organic frameworks (COFs) [20], carbon nanotube (CNT) [21], etc. Up until now, a variety of fillers have been added to prepare MMMs. For example, Sakaguchi et al. [22] successfully modified silica nanomaterials and added them to PIM-1 to fabricate MMMs. In comparison to the pure PIM-1 membrane, it showed a 6-fold increase in CO_2_ permeability (7930 Barrer) and a 15% decrease in CO_2_/N_2_ selectivity. Wu et al. [23] synthesized a covalent organic framework (SNW-1) and added it to PIM-1 to prepare MMMs. In contrast to the pure PIM-1 membrane, the CO_2_/CH_4_ and CO_2_/N_2_ selectivity of the SNW-1/PIM-1 MMMs was increased by 27.4% and 37.6%, respectively, and the CO_2_ permeability was improved by 116%.

MOFs are new porous materials, consisting of ligands and metal ions, with a large specific surface area and porosity, which become hot materials for filler in MMMs [24]. Bushell et al. [25] prepared MMMs by incorporating ZIF-8 nanoparticles as fillers into PIM-1 and investigating their permeation and sorption properties. The results showed that the CO_2_/CH_4_ selectivity of the ZIF-8/PIM-1 MMMs was clearly reduced compared with the pure PIM-1 membrane. Recently, Khdhayyer et al. [26] reported the nano MIL-101/PIM-1 MMMs. The CO_2_ permeability of the membrane increased from 5940 Barrer to 10,600 Barrer, and the CO_2_/N_2_ selectivity remained almost unchanged. The above results show that the filler can lead to significantly improved membrane permeability, but the selectivity remains almost the same, which is caused by the non-selective pores generated by the poor compatibility of the filler and PIM-1 [27]. At present, a great deal of research is focused on interfacial compatibility between polymer and filler in MMMs [28].

There are many alternatives to improve the compatibility between the polymer matrix and the MOF filler. On the one hand, surface modification of fillers or polymers is a common method to enhance the affinity between two phases in MMMs [29]. For example, Ma et al. [30] reported a polyimide/UiO-66-NH_2_ hybrid matrix membrane that performed well above the Robeson upper bound for multiple gas pairs (i.e., CO_2_/CH_4_, H_2_/CH_4_, and H_2_/N_2_). Interfacial compatibility was significantly enhanced due to hydrogen bonding between the amino group on top of UiO-66-NH_2_ and the carboxyl portion of the polymer backbone, resulting in UiO-66-NH_2_ loading of up to 55% in the membrane without introducing defects. At the same time, the gas permeability was improved by a factor of 16 compared to the pure polymer without affecting the selectivity. Furthermore, Wang et al. [31] fabricated MMMs by using PIM-1 functionalized with amidoxime groups as the matrix and MOF functionalized with amino groups as the filler. The membrane demonstrates superb separation performance with CO_2_ permeability up to 8450 Barrer, CO_2_/N_2_, and CO_2_/CH_4_ selectivity of 27.5 and 23, respectively. The hydrogen bonding between PAO-PIM-1 and UiO-66-NH_2_ tightly connects the inorganic filler to the polymer. On the other hand, the filler and the matrix can be bonded together by thermal or chemical cross-linking [32]. Experimental studies have revealed that three canoes will generate a triazine ring under heated conditions [33]. Yu et al. [34] successfully added modified UiO-66-CN to PIM-1 to prepare MMMs. After heat treatment of the membrane, UiO-66-CN and PIM-1 were cross-linked together by a triazine ring. The results showed that the membrane has excellent separation performance, with CO_2_ permeability up to 15,433 Barrer and CO_2_/N_2_ selectivity up to 27.

**Figure 1:**
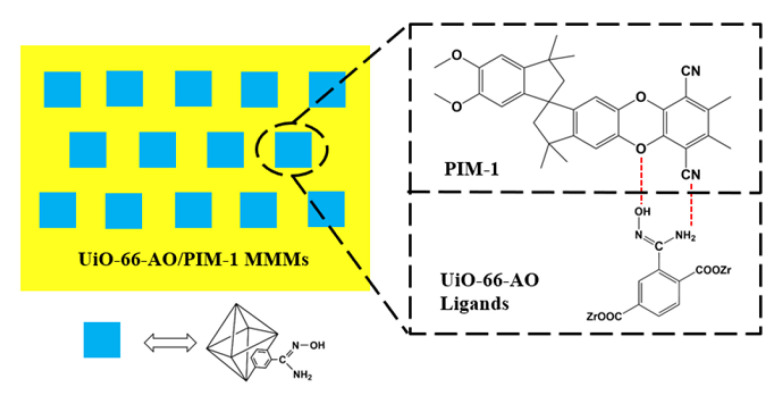
Schematic diagram of hydrogen bonding within the MMMs.

In the present work, UiO-66-AO was successfully synthesized by post-modification of UiO-66 with amidoxime and then added to the PIM-1 matrix as a filler to prepare MMMs. As shown in Figure 1, the –OH and –NH_2_ from UiO-66-AO can form hydrogen bonds with the O and N atoms above the PIM-1 chain at the interface between the matrix and the filler phase, which greatly enhances the compatibility between the filler and the polymer matrix. In addition, the selective adsorption of CO_2_ by the amidoxime group can promote the transport of CO_2_ in the membrane. The experimental results showed that the membranes exhibited better separation performance. Besides, the MMMs perform well in terms of resistance to physical aging, proving their suitability for long and continuous operation. This MMM has a bright future in gas separation thanks to its overall separation performance, which is close to the 2019 upper bound.

## 2. Materials and Methods

### 2.1. Material

5,5′,6,6′-tetrahydroxy-3,3,3′,3′-tetramethyl-1,1′-spirobisindane(TTSBI, 97%) and 2,3,5,6-tetrafluoroterephthalonitrile (TFTPN, 98%) were obtained from Shanghai yuanye Bio-Technology Co., Ltd. (Shanghai, China). N-methyl-2-pyrrolidinone (NMP, anhydrous grade, 99.8%), N, N-dimethylformamide (DMF, anhydrous grade, 99.8%), and Anhydrous potassium carbonate (K_2_CO_3_, 99.9%) were obtained from Shanghai Aladdin Biochemical Technology Co. (Shanghai, China). Toluene (C7H8, AR), chloroform (CHCl3, AR), and dichloromethane (CH_2_Cl_2_, 99.5%) were purchased from Sinopharm Chemical Reagent Co., Ltd. (Shanghai, China). Zirconium chloride (ZrCl_4_, 98%), cuprous cyanide (CuCN, 99%), and Hydroxylamine hydrochloride (HONH_2_HCl, 99%) were purchased from Shanghai Aladdin Biochemical Technology Co. (Shanghai, China). Before use, TTSBI was dissolved in methanol and reprecipitated from dichloromethane and TFTPN was vacuum sublimated.

### 2.2. Synthesis of UiO-66-CN Nanoparticles

Before synthesizing UiO-66-CN, UiO-66-Br should be synthesized. UiO-66-Br was prepared according to the method provided in the literature [35]. The first step is to add 2-Bromoterephthalic acid (556 mg, 2.27 mmol), ZrCl4 (529 mg, 2.27 mmol), benzoic acid (5.5 g, 45.0 mmol), and DMF (57 mL) to the beaker and stir the mixture at ambient temperature for 60 min. The reaction mixture was transferred to a hydrothermal kettle for reaction at 120 °C for 24 h. White precipitates were obtained by centrifugation, followed by washing with DMF (3 × 20 mL) and ethanol (3 × 20 mL), respectively, and then dried in an oven at 50 °C for 12 h in a vacuum.

Then, UiO-66-Br (0.5 g), CuCN (0.15 g), and NMP (15 mL) were put into a round-bottom flask. The original green mixture was sonicated for 20 min and heated twice in a microwave reactor for 10 min twice. Green UiO-66-CN was obtained by centrifugation, followed by washing with DMF (3 × 20 mL) and ethanol (3 × 20 mL), respectively, and then dried in an oven at 50 °C for 12 h in a vacuum.

### 2.3. Synthesis of UiO-66-AO Nanoparticles

**Figure 2:**
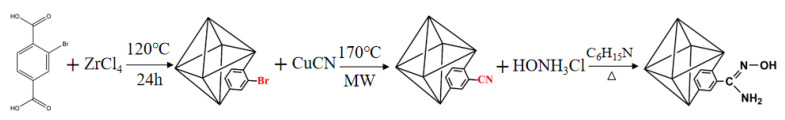
Synthetic route of UiO-66-CN and UiO-66-AO.

UiO-66-AO was synthesized based on the method in the literature [36]. UiO-66-AO (0.552 g), C_6_H_15_N (1.88 g), and HONH_3_Cl (1.29 g) were dissolved in absolute ethanol (50 mL). The mixed solution was continuously stirred for 24 h at 75 °C. The gray UiO-66-AO was acquired by washing with ethanol (3 × 20 mL) after centrifugation. The schematic diagram of the synthesis is illustrated in Figure 2.

### 2.4. Synthesis of PIM-1

PIM-1 was obtained by a high-temperature synthesis method from the literature [37,38,39]. Add TFTPN (0.01 mol, 2.0 g), TTSBI (0.01 mol, 3.4 g), anhydrous K_2_CO_3_ (0.03 mol, 4.14 g), toluene (10 mL), and DMAc (20 mL) into a 250 mL three-necked flask, respectively, and pass nitrogen into the flask. The mixture was mechanically stirred at 160 °C for 40 min. The resulting yellow, highly viscous solution was poured into methanol, and then the crude product was collected by vacuum filtration. The yellow crude product was dissolved in chloroform and reprecipitated in methanol. The final product was refluxed in water at 100 °C for 24 h and then dried at 110 °C overnight. It can be seen from the Gel Permeation Chromatography (GPC) test that PIM-1 is suitable as a membrane material with a mass average molecular weight (Mn) of 120,000 g/mol and a molecular weight distribution (PDI) of 2.01.

### 2.5. Membrane Preparation

The gas separation membrane in this experiment was prepared by the solvent evaporation method. First, PIM-1 was added to chloroform to prepare a pure PIM-1 membrane. UiO-66-AO was then added to the chloroform and stirred for 24 h. The UiO-66-AO solution was added to the PIM-1 solution in batches and continued to stir for 24 h. Finally, the mixture was poured onto the horizontal glass plate, and the solvent was slowly evaporated at room temperature for three days to get the MMM. The thickness of the membrane is measured with a digital display thickness gauge. The thickness of the membrane is the average of the results measured at multiple locations. The membrane thickness is about 70 μm. In order to distinguish the types of MMMs, the membrane is uniformly defined as UiO-66-AO@PIM-1 X%, where X represents the mass fraction of UiO-66-AO.

### 2.6. Characterization

MOF chemical structure was analyzed by Fourier infrared spectroscopy (FI-TR, Bruker, Mannheim, Germany) with a scan range of 4000–400 cm^−1^. The MOF crystal structure was analyzed by X-ray diffraction (XRD, Rigaku Corporation SmarLab, Tokyo, Japan) with a scan angle of 5–50 and a scan rate of 5°/min. The micromorphology of MOF was observed by scanning electron microscopy (SEM, JSM-7601F, Tokyo, Japan). The thermal stability of MOF was tested by thermogravimetric analysis (TGA, TAQ500, New Castle, DE, USA) at a temperature increase rate of 10 °C/min in the range of 25–800 °C. Specific surface area and pore volume were determined by the nitrogen adsorption-desorption experiment with Micrometrics ASAP 2020 at 77 K.

### 2.7. Gas Separation Experiment

In this experiment, a self-made gas penetration test device was used. Gas flux tests were performed using the vacuum method to test three pure gases: high purity N_2_, high purity CH_4_, and high purity CO_2_, respectively. Three pure gases were tested at 25 °C and 2 bar. The formula for calculating gas permeability is as follows:

(1) $$P=\frac{V_{d}l}{p_{2}ART}\left[{\left(\frac{dp_{1}}{dt}\right)}_{ss}-{\left(\frac{dp_{1}}{dt}\right)}_{leak}\right]$$

where *P* is the membrane permeability in Barrer (1 Barrer = 10^−10^ cm^−3^ (STP) cm cm^−3^ s^−1^ cmHg^−1^). *V_d_* represents the permeate side volume (cm^3^). *l* represents the membrane thickness (cm). $p_{2}$ represents the raw side absolute pressure (cmHg). *A* represents the effective sample area of the membrane (cm^2^). *R* represents the gas constant with a value of 0.278 cmHg cm^3^ cm^−3^ (STP) K^−1^. *T* represents the room temperature (K), which is constant at 298.15 K. ${\left(dp_{1}/dt\right)}_{ss}$ and ${\left(dp_{1}/dt\right)}_{leak}$ are the rates of pressure rise (cmHg s^−1^) on the permeate side under test pressure and under vacuum, respectively. The ideal selectivity (α) is calculated by the following equation:

(2) $$\alpha (x/y)=\frac{P_{x}}{P_{y}}$$

Solubility coefficient (*S*) and Diffusion coefficient (*D*) can be calculated as follows:

(3) $$D=\frac{l^{2}}{6\theta }$$

(4) $$S=\frac{P}{D}$$

where *x* and *y* are different gas composition, θ represents the lag time.

## 3. Results

### 3.1. Characterization of UiO-66-CN and UiO-66-AO

**Figure 3:**
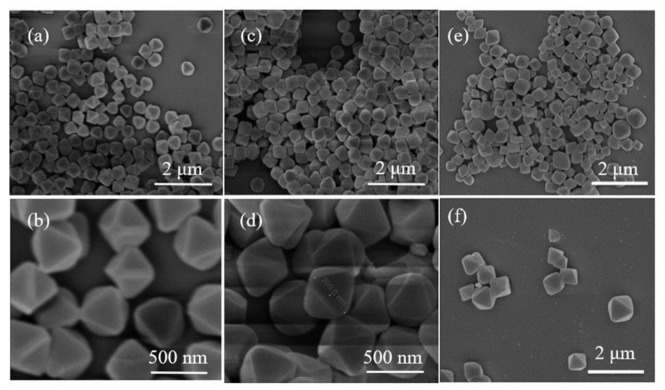
SEM pictures: (**a**,**b**) UiO-66-Br; (**c**,**d**) UiO-66-CN; (**e**,**f**) UiO-66-AO.

**Figure 4:**
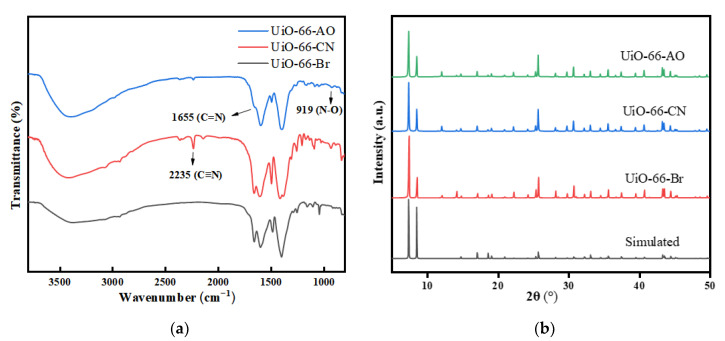
Characterization of the chemical structure of UiO-66-AO: (**a**) FT-IR spectra; (**b**) XRD pattern.

**Figure 5:**
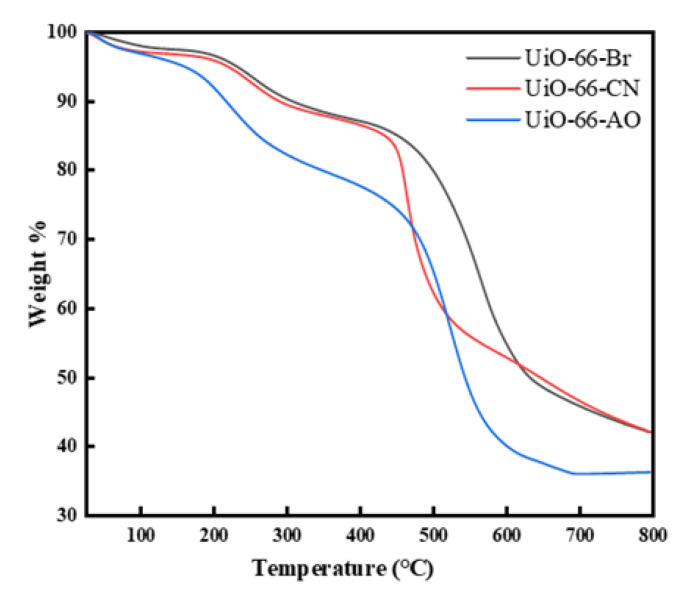
TGA curves of UiO-66-AO.

The SEM images of UiO-66-CN and UiO-66-AO powders are shown in Figure 3. Compared with UiO-66-Br, the crystal shape after modification has not changed, and the size is uniform at about 350 nm, which is suitable for the filler of MMMs. Figure 4a is the FTIR spectrum of UiO-66-CN and UiO-66-AO. For UiO-66-CN, the absorption peaks at 1400, 1580, and 1655 cm^−1^ are characteristic peaks of –C=O–O [40]. The peak at 2235 cm^−1^ is the characteristic peak of –CN. In the UiO-66-AO spectrum, the peak at 2235 cm^−1^ disappears significantly and new characteristic peaks appear at 1655 cm^−1^ and 919 cm^−1^. 1655 cm^−1^ is caused by the stretching vibration of C=N and 919 cm^−1^ is the result of the N-O bending vibration [41].The XRD diffraction patterns of UiO-66-CN and UiO-66-AO are shown in Figure 4b. The positions of the diffraction peaks of the two powders are consistent with those of simulated UiO-66, indicating that they have the same crystal structure [42]. No other peaks were found in the spectra of UiO-66-AO and UiO-66-CN, indicating that the purity was high and that the post-modification did not change the original skeleton structure of UiO-66-AO and UiO-66-CN. This shows that the cyano group was successfully replaced by the amidoxime group. Figure 5 shows the TGA curves of UiO-66-CN and UiO-66-AO. The weight loss of the three nanocrystals can be divided into three steps. First of all, it experienced a small mass loss in the range of 0–200 °C, which was mainly caused by the residual or absorbed water in the drug pores. Then, the mass loss in the range of 200–400 °C can be attributed to the thermal decomposition of –Br, –CN and –CNH_2_NOH. Finally, the crystal structure will collapse when the temperature exceeds 500 °C.

### 3.2. Membrane Characterization

#### 3.2.1. Scanning Electron Microscope (SEM)

**Figure 6:**
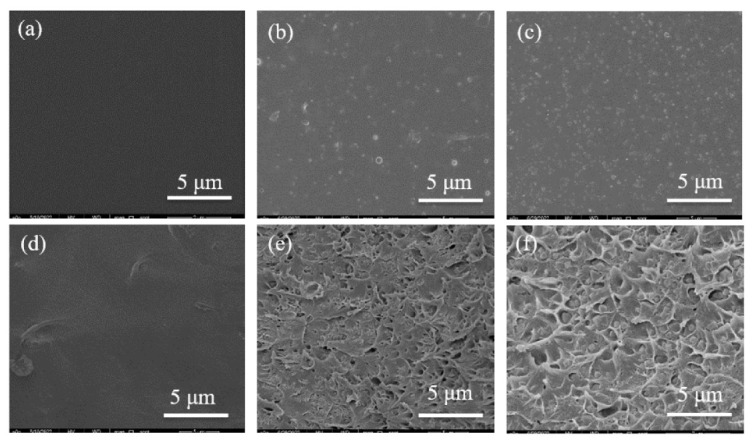
Surface and cross-sectional SEM images of UiO-66-AO@PIM-1 MMMs with different loadings: (**a**,**d**) 0 wt%; (**b**,**e**) 10 wt%; (**c**,**f**) 30 wt%.

The microstructure of the membrane was clearly analyzed by SEM. The partial surface and cross-section of the UiO-66-AO@PIM-1 MMMs are shown in Figure 6. As can be seen from the surface diagram of the membrane, the surface is dense, smooth, and free of defects. With the increase in filler loading, more and more white particles gradually appear. The white particles are uniformly dispersed on the membrane surface, and no cavities are found around them, which indicates good compatibility between the filler and the polymer. In the cross-section of the membrane, nanoparticles can be clearly seen in the MMMs, which can prove that UiO-66-AO was successfully dispersed in PIM-1. As the loading increased, many crescent-shaped protrusions were produced on the cross-section. This may be caused by the phase separation due to the filler addition. At 30 wt% loading, there was also no agglomeration, which proves the good dispersion of UiO-66-AO in the membrane. This also indicates that the modified UiO-66-AO plays an important role in improving the interfacial compatibility problem.

#### 3.2.2. X-ray Powder Diffraction (XRD)

**Figure 7:**
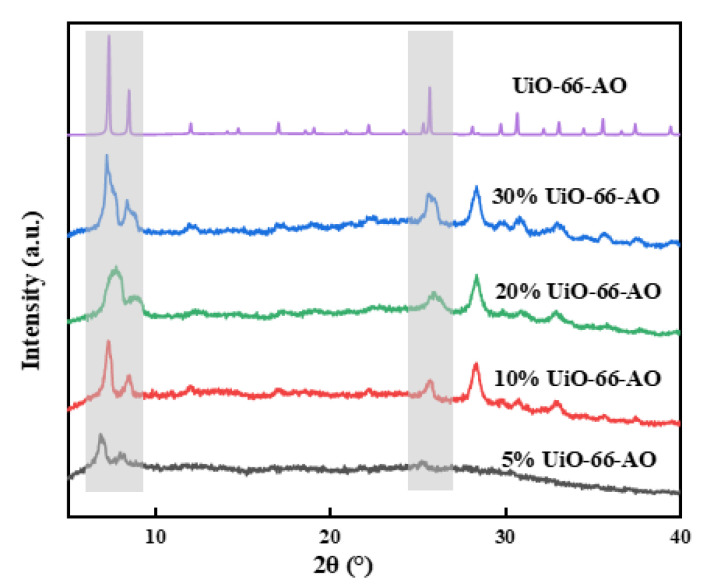
XRD spectra of UiO-66-AO@PIM-1 MMMs.

Figure 7 presents the XRD spectra of the membranes with different loadings. UiO-66-AO is highly crystalline and has a correct crystal structure, with characteristic diffraction peaks at 2θ of 7.29°, 8.44° and 25.48° that represent the (111), (200), and (600) crystal planes, respectively. The characteristic diffraction peaks of UiO-66-AO appeared in all the MMMs and became stronger with increasing loading. This proves that the crystal structure of UiO-66-AO did not change after membrane preparation and that it can exist stably in the MMMs.

#### 3.2.3. Fourier Transform Infrared Spectrometer (FT-IR)

**Figure 8:**
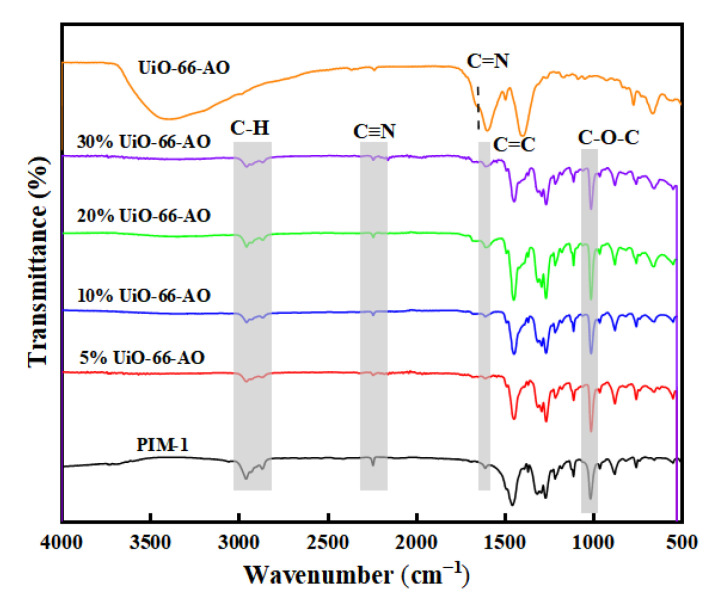
XRD spectra of UiO-66-AO@PIM-1 MMMs.

The chemical structure of the membrane was analyzed by FT-IR, as shown in Figure 8. The spectrogram of the pure PIM-1 membrane is consistent with the previous report [33]. The peaks at 2853–2954 cm^−1^ are the absorption peaks of the C–H bond on the benzene ring and the methyl group on the five-membered ring. The characteristic peak at 2238 cm^−1^ represents the cyano group on the benzene ring. The characteristic peak at 1600 cm^−1^ is the C=C bond on the benzene ring. The characteristic peak at 1005 cm^−1^ is the C–O–C group on the six-membered ring attached to the two benzene rings. With the filler content increasing, the intensity of the peak of cyano weakened significantly, and the intensity of the C=N peak of the amidoxime group gradually strengthened, which proved that the chemical structure of UiO-66-AO existed stably in the membranes. Moreover, no other characteristic peaks were found in the UiO-66-AO@PIM-1 MMM, demonstrating that there is no strong interaction between UiO-66-AO and PIM-1.

#### 3.2.4. Thermogravimetric Analysis (TGA)

**Figure 9:**
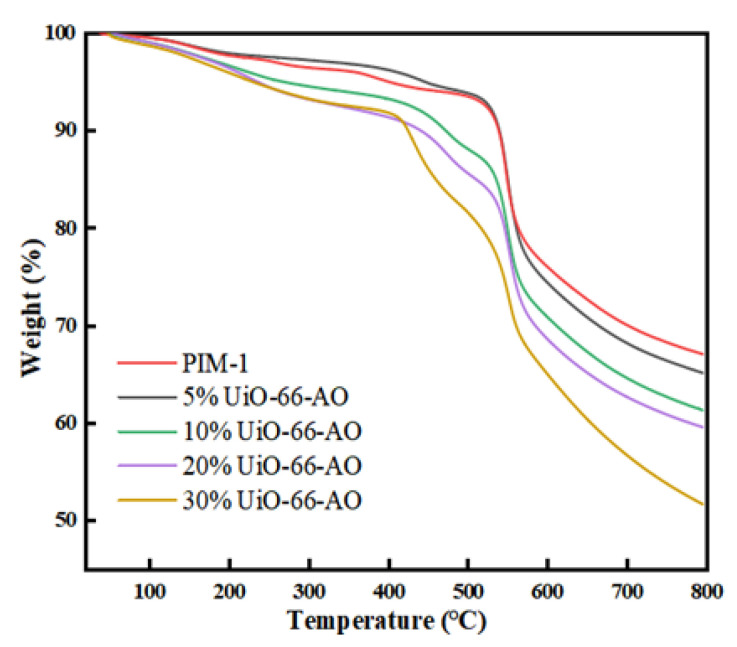
TGA curves of UiO-66-AO@PIM-1 MMMs.

The thermal stability of UiO-66-AO@PIM-1 MMMs was investigated by TGA (Figure 9). Pure PIM-1 membrane has a small mass loss until 400 °C, indicating its high thermal stability. When nanofillers were added to the membrane, the curves of MMMs were still consistent with that of pure PIM-1 membrane. The thermal stability of the MMMs showed some decrease, but the overall decomposition temperature was still higher than 400 °C. The thermal decomposition curve of the MMMs can be divided into several parts. The weight loss until 160 °C can be attributed to the water inside the membrane and the solvent in the pores of UiO-66-AO. The weight loss from 160 °C to 400 °C is caused by the decomposition of the polymer monomer and amidoxime on UiO-66-AO. When the temperature is higher than 400 °C, the main chain of polymer PIM-1 starts to decompose and the UiO-66-AO skeleton gradually collapses.

### 3.3. Gas Permeation Experiment

The gas separation performance was tested at 2 bar and 25 °C with pure CO_2_, N_2_, and CH_4_. The permeation process of gas molecules through gas separation membranes can be explained by the dissolution-diffusion model. Usually, the adsorption-desorption of gases on the membrane surface proceeds faster, and diffusion in the membrane is slower. However, the diffusion of gas molecules in the membrane is usually negatively correlated with their molecular kinetic diameters, which are 3.3 Å, 3.64 Å, and 3.8 Å for CO_2_, N_2_, and CH_4_, respectively [43]. The higher permeability of CH_4_ than N_2_ is due to the much higher solubility of CH_4_ than N_2_. The gas separation performance data of the UiO-66-CN/PIM-1 and UiO-66-AO@PIM-1 MMMs are presented in Table S1 and Table 1. The permeability of the three gases increases simultaneously with the loading of the filler. On the one hand, the dynamic diameters of the gases are smaller than the pore diameter of UiO-66-CN, which enables the gas molecules to pass through faster. Compared with the distorted structure of the PIM-1 membrane, the gas can pass through the straight channels in UiO-66-CN. On the other hand, the addition of UiO-66-CN also increases the free volume of the membrane, thus improving the gas permeability. The CO_2_/N_2_ and CO_2_/CH_4_ selectivity increase to their highest levels with the increase in UiO-66-CN loading and then gradually decrease. It is well known that UiO-66 is a common adsorbent material. Similarly, the metal clusters of UiO-66-CN can selectively adsorb CO_2_. In addition, smaller CO_2_ molecules can pass through the internal pore channels of UiO-66-CN more rapidly. When the loading of UiO-66-CN is too high, it is difficult to completely coat the surface of the filler due to the small amount of polymer, which will create non-selective pore channels at the interface of the two phases and cause a decrease in the selectivity of the membrane. Compared with the previous UiO-66/PIM-1 MMMs in the literature, the selectivity of the UiO-66-CN/PIM-1 MMMs was slightly improved due to the enhanced adsorption of CO_2_ by the N atoms on the cyano. We further modified –CN to an amidoxime group to obtain a new UiO-66-AO. The permeability of the UiO-66-AO@PIM-1 MMMs increases with increasing filler loading, and the selectivity gradually increases and then decreases. The difference is that there is an overall enhancement in the selectivity of the gas pair. The CO_2_/N_2_ selectivity of UiO-66-AO@PIM-1 MMMs is improved from 22.4 to 26.9, and the CO_2_/CH_4_ selectivity is improved from 15.9 to 16.5 compared to UiO-66-CN/PIM-1 MMMs. A large amount of –NH_2_, –OH on UiO-66-AO and O, N atoms on the PIM-1 chain form hydrogen bonding structures, which can greatly improve the interfacial compatibility and enhance the selectivity in the membrane.

**Figure 10:**
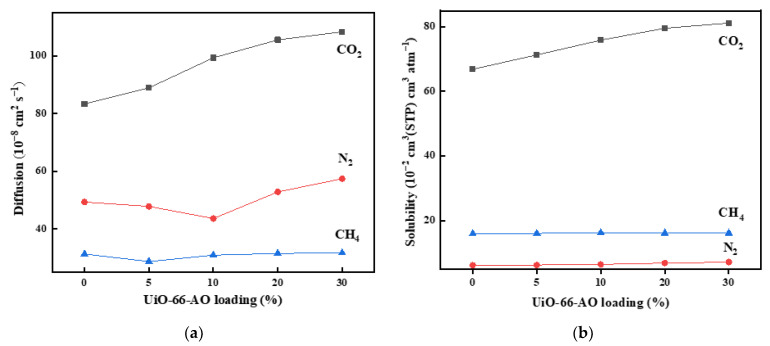
Diffusivity coefficients (**a**) and solubility coefficients (**b**) of UiO-66-AO@PIM-1 MMMs.

**Figure 11:**
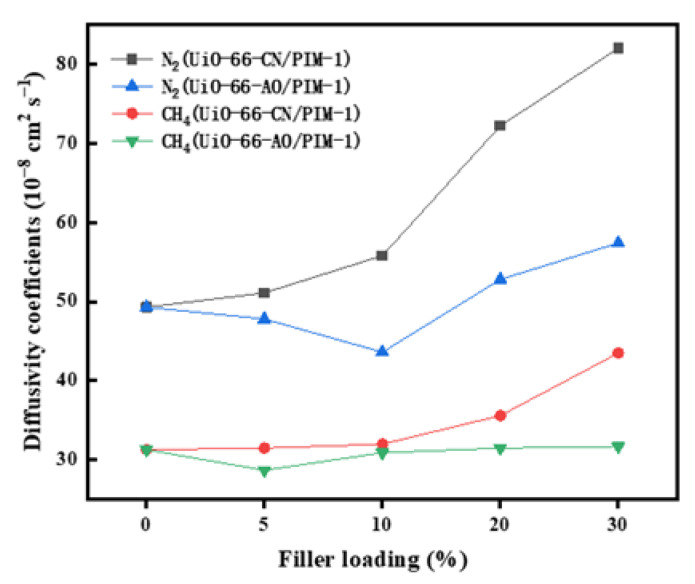
Comparison of N2 and CH4 diffusion coefficients of UiO-66-CN and UiO-66-AO based MMMs.

To further investigate the gas permeation mechanism and the structure of the membrane, we calculated the solubility coefficient (S) and diffusion coefficient (D) of the membrane by the time lag method. The diffusivity and solubility coefficients of MMMs with different loadings are shown in Tables S2 and S3. As shown in Figure 10, the diffusivity coefficients of the three gases incrementally improved as the UiO-66-AO loading increased from 0 to 30 wt%. There is a higher free volume fraction and more transmission pathways after UiO-66-AO is added to the membrane. The solubility coefficients of CO_2_ remarkably increased with the loading, while the solubility coefficients of N_2_ and CH_4_ changed slightly, mainly due to the selective adsorption of CO_2_ by UiO-66-AO. When the UiO-66-AO loading was changed from 10% to 30%, the increment of N_2_ and CH_4_ diffusion coefficients got significantly larger, which proved that the filler and polymer could not be well combined, resulting in large non-selective cavitation. At 10% of the identical loading, the UiO-66-AO@PIM-1 MMMs and the UiO-66-CN/PIM-1 MMMs had similar CO_2_ permeability, but the CO_2_/N_2_ selectivity improved from 22.4 to 26.9. Besides, from Figure 11, the diffusivity coefficients of N_2_ and CH_4_ of the UiO-66-AO@PIM-1 MMMs became apparently smaller, confirming that the UiO-66-AO@PIM-1 MMMs greatly improved the interfacial compatibility and reduced the non-selective pore channels.

### 3.4. Anti-Aging Properties

**Figure 12:**
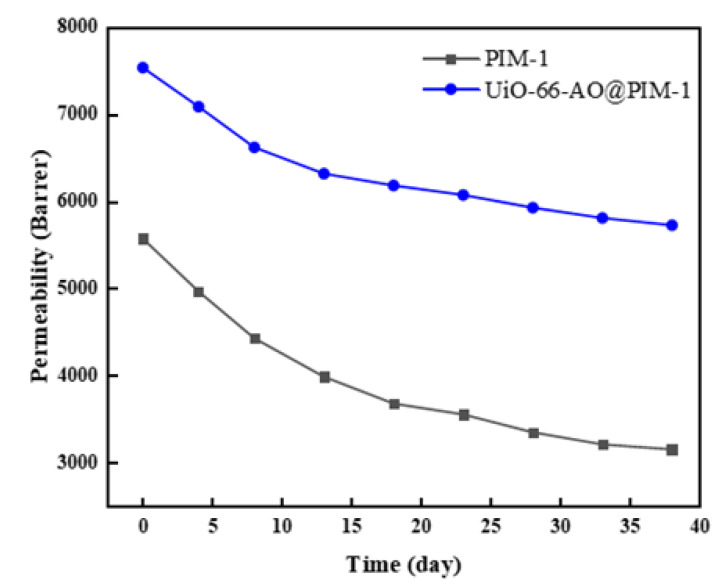
Physical aging test of PIM-1 and UiO-66-AO@PIM-1 membrane for CO_2_.

Physical aging is a common problem with PIM membranes, which can lead to a loss of membrane permeability over time [44]. Here we investigated the anti-aging effect after adding UiO-66-AO to the PIM-1 membrane. We put the membrane in a cool and dry place and tested its permeability every 3 days. As we can see in Figure 12, after 38 days of aging, the CO_2_ permeability of the pure PIM-1 membrane decreased by 43.4%; however, the CO_2_ permeability of the 10% UiO-66-AO@PIM-1 membrane decreased by merely 22.0%, mainly due to the addition of porous filler, which limits the free motion of polymer chains and prevents the collapse of PIM-1 micropores, so the membranes maintain a high free volume. There is better interaction between UiO-66-AO and PIM-1 molecular chains, which fixes the molecular chains, so UiO-66-AO@PIM-1 membranes show better anti-aging effects. Above all, the inorganic filler had an excellent effect on improving the aging problem of the membrane, and the addition of modified UiO-66-AO played an eminent role in enhancing the anti-aging effect of PIM-1 membranes.

### 3.5. Comparison with Literature

**Figure 13:**
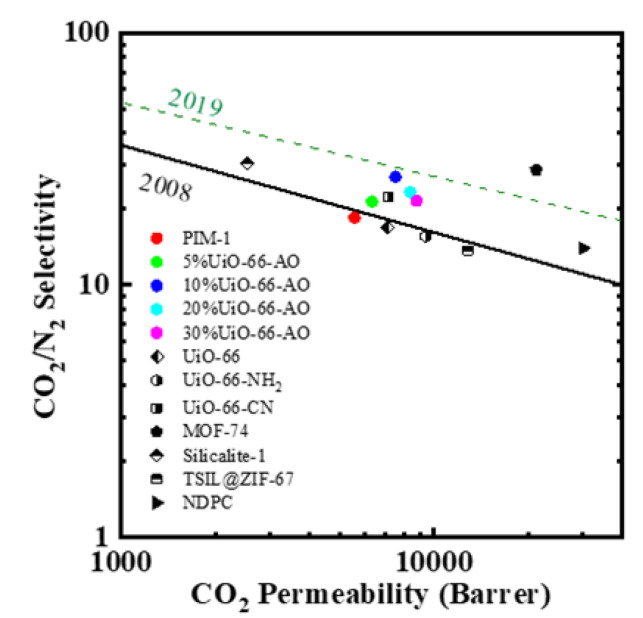
Comparison of CO_2_/N_2_ separation performance between our work and other MMMs (UiO-66/PIM-1 [45], UiO-66-NH_2_/PIM-1 [31], MOF-74/PIM-1 [32], Silicalite-1/PIM-1 [46], TSIL@ZIF-67/PIM-1 [47], NDPC/PIM-1 [48]).

Significantly, the CO_2_/N_2_ separation performance of the UiO-66-AO@PIM-1 MMMs was close to the 2019 upper bound (Figure 13). In addition, compared with other PIM-1 membranes, the UiO-66-AO@PIM-1 MMMs exhibited even better separation performance. This is further evidence that UiO-66-AO plays an instrumental role in the overall performance of PIM-1 membranes.

## 4. Discussion

By modifying UiO-66 with an amoxime group, not only is the interfacial compatibility of the MMMs enhanced, but a large number of pro-CO_2_ groups are also introduced, which synergistically optimize the solution and diffusion processes of the gas in the membrane. However, the compatibility and aging problems need to be further improved, which can be achieved by cross-linking the filler and matrix together directly through a chemical reaction.

## 5. Conclusions

In this work, a novel gas separation membrane was fabricated with high CO_2_ permeability and selectivity by incorporating UiO-66-AO in PIM-1 polymer. UiO-66-AO was synthesized by post-modification and used for the first time in CO_2_ separation. UiO-66-AO can maintain the high porosity, thermal stability, and high crystallinity of UiO-66. The UiO-66-AO@PIM-1 MMMs with 10 wt% loadings exhibit excellent gas separation performance. Compared to the pure PIM-1 membrane, the CO_2_ permeability improved by 60%, and the CO_2_/N_2_ selectivity improved by 30%. Compared to the UiO-66-CN/PIM-1 membrane, the permeability remained almost unchanged, but the CO_2_/N_2_ selectivity increased from 22.4 to 26.9. The interfacial compatibility is significantly enhanced due to the direct hydrogen bonding between the amidoxime-functionalized MOFs and the polymer backbone, leading to increased selectivity. Furthermore, the UiO-66-AO@PIM-1 MMM also exhibits excellent anti-aging effects, mainly attributed to the incorporated nanoparticles UiO-66-AO. The strategy of constructing hydrogen bonds between the filler and polymer phase interfaces using post-filler modification is an effective way to improve the interfacial defects of the two phases in the membrane.

## Figures and Tables

**Table 1:** Averaged permeabilities and selectivities at 25 °C and 2 bar, for the pristine PIM-1 and the derived MMMs.

UiO-66-AO	Permeability (P) (Barrer)			Ideal Selectivity (*α*)	
	CO_2_	N_2_	CH_4_	CO_2_/N_2_	CO_2_/CH_4_
0	5573.24	300.97	496.18	18.52	11.23
5%	6335.77	296.06	458.45	21.44	13.82
10%	7535.54	279.24	500.29	26.90	15.06
20%	8398.75	358.77	506.75	23.41	16.57
30%	8792.33	407.86	509.57	21.56	15.25

## Supplementary Materials

The following supporting information can be downloaded at: https://www.mdpi.com/article/10.3390/membranes13090781/s1, Figure S1: Chemical structure characterization of PIM-1: (a) FT-IR spectra; (b) 1H NMR spectra; Figure S2: Surface and cross-sectional SEM images of UiO-66-AO@PIM-1 MMMs with different loadings: (a,b) 0 wt%; (c,d) 10 wt%; (e,f) 10 wt%; (g,h) 20 wt%; (i,j) 30 wt%; Table S1: Averaged permeabilities and selectivities at 25 °C and 2 bar, for the pristine PIM-1 and the UiO-66-CN MMMs; Table S2: Diffusivity (10^−8^ cm^2^ s^−1^) and solubility (10^−2^ cm^3^(STP) cm^3^ atm^−1^) coefficients, diffusivity selectivity (α_D_) and solubility selectivity (α_S_) for UiO-66-CN/PIM-1 MMMs; Table S3: Diffusivity (10^−8^ cm^2^ s^−1^) and solubility (10^−2^ cm^3^ (STP) cm^3^ atm^−1^) coefficients, diffusivity selectivity (α_D_) and solubility selectivity (α_S_) for UiO-66-AO@PIM-1 MMMs.
