# Supplementary material for: Amidoxime Modified UiO-66@PIM-1 Mixed-Matrix Membranes to Enhance CO2 Separation and Anti-Aging Performance
Source: Membranes (Basel). 2023 Sep 6;13(9):781. doi: 10.3390/membranes13090781 (PMC10536640; doi:10.3390/membranes13090781)
Supplement: Supplementary file 1 [file membranes-13-00781-s001.zip › membranes-2509156-supplementary.pdf]

Supplementary Information

Amidoxime Modified UiO-66@PIM-1 Mixed-Matrix Membranes to Enhance CO_2 Separation and Anti-Aging Performance

Jiaming Gao [1], Yongchao Sun [1], Feifei Kang [1], Fei Guo [1], Gaohong He [1], Hanli Wang [2], Zhendong Yang [2], Canghai Ma [1], Xiaobin Jiang [1] and Wu Xiao [1,*]

[1] State Key Laboratory of Fine Chemicals, Frontier Science Center for Smart Materials, Dalian University of Technology, Dalian 116024, China; jiaminggao97@outlook.com (J.G.); yongchao_sun@mail.dlut.edu.cn (Y.S.); kangfeifei1998@outlook.com (F.K.); guofei@mail.dlut.edu.cn (F.G.); hgaohong@dlut.edu.cn (G.H.); cma@dlut.edu.cn (C.M.); xbjiang@dlut.edu.cn (X.J.)

[2] Shandong Huaxia Shenzhou New Material Co., Ltd., Zibo 256401, China; whl89333@huaxiashenzhou.com (H.W.); yangzhendong@huaxiashenzhou.com (Z.Y.)

* Correspondence: wuxiao@dlut.edu.cn

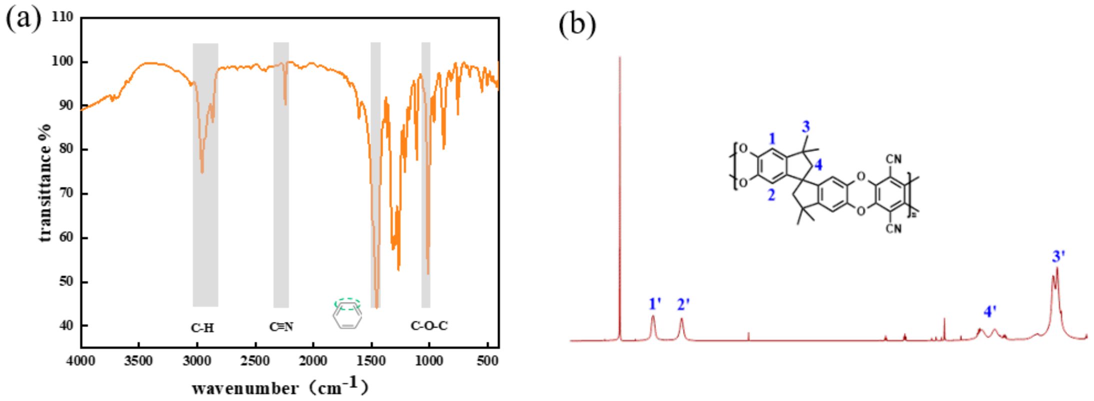

Figure S1. Chemical structure characterization of PIM-1: (**a**) FT-IR spectra; (**b**) ^{1}H NMR spectra.

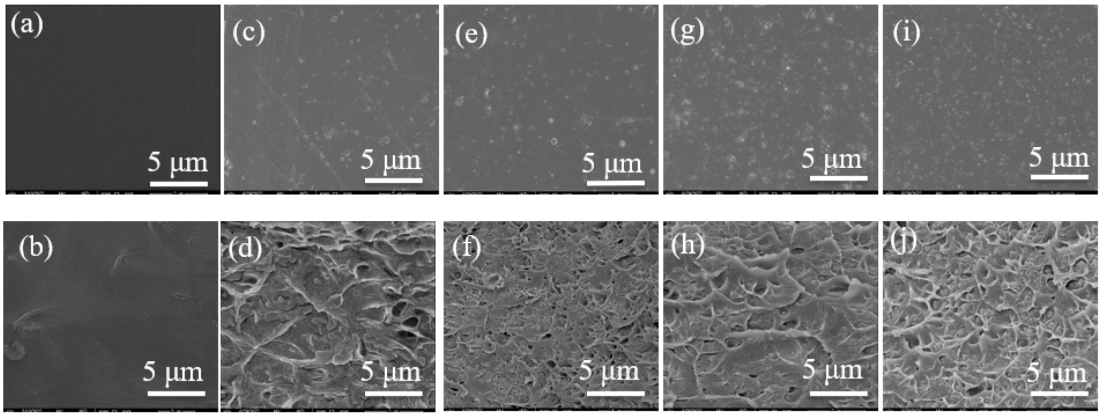

Figure S2. Surface and cross-sectional SEM images of UiO-66-AO@PIM-1 MMMs with different loadings: (**a**,**b**) 0 wt%; (**c**,**d**) 10 wt%; (**e**,**f**) 10 wt%;(**g**,**h**) 20 wt%; (**i**,**j**) 30 wt%

Table S1. Averaged permeabilities and selectivities at 25 °C and 2 bar, for the pristine PIM-1 and the UiO-66-CN MMMs.

UiO-66-CN	Permeability (P) (Barrer)			Ideal selectivity (α)	
	CO_2	N_2	CH_4	CO_2/N_2	CO_2/CH_4
0	5573.24	300.97	496.18	18.52	11.23
5 wt%	6288.54	312.39	504.69	20.13	12.46
10 wt%	7135.02	318.49	515.50	22.40	13.84
20 wt%	9027.93	468.47	566.72	19.23	15.93
30 wt%	9965.11	549.63	704.23	18.13	14.15

Table S2. Diffusivity (10^{-8} cm^2 s^{-1}) and solubility (10^{-2} cm^3(STP) cm^3 atm^{-1}) coefficients, diffusivity selectivity (α_D) and solubility selectivity (α_S) for UiO-66-CN/PIM-1 MMMs.

UiO-66-CN loading	Diffusivity			Solubility			α_D		α_S	
	CO_2	N_2	CH_4	CO_2	N_2	CH_4	CO_2/N_2	CO_2/CH_4	CO_2/N_2	CO_2/CH_4
0%	83.3	49.3	31.3	66.9	6.1	15.9	1.7	2.7	11.0	4.2
5%	89.2	51.1	31.5	70.5	6.1	16.0	1.8	2.8	11.2	4.4
10%	93.1	55.8	32.0	76.7	5.7	16.1	1.9	2.9	11.8	4.8
20%	111.7	72.2	35.6	80.8	6.4	15.9	1.5	3.1	12.6	5.1
30%	119.9	82.0	43.5	83.1	6.7	16.2	1.4	2.8	12.5	5.1

Table S3. Diffusivity (10^{-8} cm^2 s^{-1}) and solubility (10^{-2} cm^3(STP) cm^3 atm^{-1}) coefficients, diffusivity selectivity (α_D) and solubility selectivity (α_S) for UiO-66-AO@PIM-1 MMMs.

UiO-66-AO loading	Diffusivity			Solubility			α_D		α_S	
	CO_2	N_2	CH_4	CO_2	N_2	CH_4	CO_2/N_2	CO_2/CH_4	CO_2/N_2	CO_2/CH_4
0%	83.3	49.3	31.3	63.9	6.1	15.9	1.7	2.7	11.0	4.2
5%	88.9	47.8	28.7	71.3	6.2	16.0	1.9	3.1	11.5	4.5
10%	99.3	43.6	30.9	75.9	6.4	16.2	2.3	3.2	11.9	4.7
20%	105.5	52.8	31.5	79.6	6.8	16.1	2.0	3.4	11.7	4.9
30%	108.3	57.4	31.7	81.2	7.1	16.1	1.9	3.3	11.4	5.1
